# C-Reactive Protein/Albumin Ratio and Clinicopathological Features in Ovarian Cancer: A Prospective Study

**DOI:** 10.7759/cureus.76542

**Published:** 2024-12-28

**Authors:** Archana Barik, Apoorva Rashmi, Vinita Singh, Amitabh Kumar Upadhyay

**Affiliations:** 1 Obstetrics and Gynaecology, Manipal Tata Medical College, Jamshedpur, IND; 2 Obstetrics and Gynaecology, Tata Main Hospital, Jamshedpur, IND; 3 Obstetrics and Gynaecology, Jayshree Multispeciality Hospital, Bhachau, IND; 4 Medical Oncology, Tata Main Hospital, Jamshedpur, IND

**Keywords:** clinicopathological features, c-reactive protein (crp), crp/alb ratio, ovarian cancer, serum albumin (alb)

## Abstract

Introduction

Evidence suggests inflammation plays a key role in the development of ovarian malignancy. This study investigated the relationship between the C-reactive protein (CRP) to serum albumin (Alb) ratio and clinicopathological parameters in ovarian cancer patients. The goal was to determine if this readily measurable inflammatory marker could provide insights into disease severity.

Methods

A prospective study of 94 patients with histopathologically confirmed ovarian cancer (diagnosed between November 2020 and December 2023) investigated the relationship between the pretreatment C-reactive protein to albumin ratio (CRP/Alb), and various clinicopathological characteristics, including age, distant metastasis, lymph node involvement, ascites, tumor grade, and surgical stage. Receiver operating characteristic (ROC) curve analysis was used to assess the diagnostic accuracy of the CRP/Alb ratio and establish a cutoff value.

Results

Patients with metastasis, lymph node involvement, ascites, higher tumor grade, and advanced stages (III and IV) had significantly higher median CRP/Alb ratios compared to those without these features (1.43 vs. 0.78, 1.47 vs. 0.51, 1.16 vs. 0.46, 1.46 vs. 0.52, and stages I/II (0.49/0.95) vs. stages III/IV (1.47/1.58), respectively; all p < 0.001). ROC analysis determined an optimal cutoff value of 1.08. A preoperative CRP/Alb ratio above this cutoff indicated severe disease based on clinicopathological parameters.

Conclusion

In conclusion, elevated pretreatment CRP/Alb ratios correlated with more severe and advanced ovarian cancer.

## Introduction

Ovarian cancer, the third most common gynecological cancer globally, is a leading cause of female morbidity and mortality [1]. In India, a population-based cancer registry reported a crude incidence rate of 5.08 ovarian cancers per 100,000 individuals and a crude mortality rate of 2.3 per 100,000 [2]. Growing evidence implicates systemic inflammation in its carcinogenesis and progression. The inflammation hypothesis is supported by several observations. The ovulatory process itself is inherently inflammatory and considered as a risk factor for ovarian cancer [3]. The repeated cycles of inflammation associated with ovulation may contribute to the cumulative damage that leads to cancer development. Certain conditions associated with pelvic inflammation, such as endometriosis and polycystic ovarian syndrome (PCOS), are associated with a heightened risk of ovarian cancer [3]. These conditions create a persistent inflammatory environment within the pelvis, potentially promoting cancerous changes in ovarian tissue. The hypothesis is further bolstered by the fact that interventions that reduce ovarian cancer risk appear to act by mitigating inflammation. Oral contraceptive pills, for example, suppress ovulation and thus reduce the inflammatory burden. Similarly, tubal ligation and hysterectomy can lower risk, potentially by preventing the ascent of pro-inflammatory factors from the lower genital tract to the ovaries [4]. Furthermore, studies have demonstrated a potential protective effect from the use of anti-inflammatory medications, supporting the link between inflammation and ovarian cancer development [5].

Poor prognosis in numerous cancers, including ovarian cancer, is strongly linked to systemic inflammation, as evidenced by prognostic scores such as the Glasgow Prognostic Score (GPS), its modified versions (mGPS and HS-mGPS), neutrophil-lymphocyte ratio (NLR), platelet-lymphocyte ratio (PLR), and systemic immune-inflammation index (SII) [6]. C-reactive protein (CRP), a key acute-phase reactant, independently predicts prognosis in many malignancies; its elevation accelerates angiogenesis through increased vascular growth factors and interleukins, thereby promoting tumor progression and decreased survival [7-9]. Furthermore, hypoalbuminemia, often reflecting poor nutrition and sustained systemic inflammatory response syndrome (SIRS), is also associated with adverse outcomes in various cancers [10-12]. Consequently, the CRP and serum albumin ratio (CRP/Alb), a readily available marker reflecting inflammatory status, serves as an attractive indicator of cancer severity and prognosis, demonstrating prognostic value in several cancers, including hepatocellular, gastric, and esophageal squamous cell carcinoma [13-16]. Although some studies suggest a positive correlation between this ratio and ovarian cancer severity, evidence remains geographically limited [17,18]. This study, undertaken at a tertiary care hospital in eastern India, aims to further investigate the association between the CRP/Alb ratio and the clinicopathological parameters of ovarian cancer.

## Materials and methods

Informed consent was obtained from all study participants before recruitment, and the study received ethical approval from the Institutional Ethical Committee of Tata Main Hospital, Jamshedpur, Jharkhand, India. This prospective observational study was conducted from November 2020 to December 2023 at a tertiary care hospital (Tata Main Hospital, Jamshedpur) of Jharkhand state in the eastern part of India. The study included all patients with histopathologically confirmed ovarian cancer (inclusion criteria), excluding those with borderline ovarian tumors, multiple malignancies, infections, or inflammatory conditions (exclusion criteria). Ninety-four patients were enrolled using convenience sampling from the gynecology outpatient department (OPD) in the allotted study period. Complete blood counts, tumor markers, C-reactive protein (CRP), and serum albumin levels were evaluated, along with computed tomography (CT) scans (abdomen, pelvis, and thorax), ultrasound (abdomen and pelvis) and histopathological report of the tumor mass. Patients received treatment appropriate to their disease status. Moreover, all the patients underwent surgical staging. Chemotherapy was guided by the histopathological results. The study aimed to determine the association between the CRP/Alb ratio and clinicopathological parameters, including age, tumor stage (FIGO staging system: International Federation of Gynecology and Obstetrics staging system) and grade, histological type, ascites, lymph node involvement, Cancer antigen 125 (CA-125) levels, and metastasis in ovarian cancer patients.

Data collection procedure

Eligible participants were screened and selected from the Gynecology OPD. The principal investigator or co-investigator interviewed the selected patients. Information on demographic data, clinical history, and investigations were noted down using a preformatted questionnaire (Appendices). All participants were followed up until treatment completion.

Statistical analysis

This study utilized Microsoft Excel 365 for data collection. Continuous variables were summarized using means ± standard deviations, medians with interquartile ranges (IQRs), and ranges. Categorical variables were presented as counts and percentages. The chi-square test or Fisher's exact test was employed to assess the association between categorical variables. A receiver operating characteristic (ROC) curve analysis was performed to evaluate the diagnostic accuracy of both the CRP/Alb ratio and CA-125. The Youden index was used to determine the optimal cutoff point for the CRP/Alb ratio, which was then used to categorize patients into distinct groups. The Mann-Whitney U test was employed to find out the association of CRP/Alb ratio and CA-125 with the clinicopathological parameters. All statistical tests were two-sided, with a significance level set at p < 0.05. Data analysis was conducted using SPSS Version 28.0 (IBM Corp., Armonk, NY, USA).

## Results

A total of 94 patients were evaluated with a median age of 51 years (range of 12-85 years). The median body mass index (BMI) was 22 (kg/m^2^), ranging from 17 to 26.2 (kg/m^2^). Table 1 shows the demographic and other characteristics of the study population.

**Table 1:** Demographic and Other Characteristics of Study Population SD: Standard Deviation; IQR: Interquartile Range; Min: Minimum; Max: Maximum; BMI: Body Mass Index; CRP/Alb Ratio: C-reactive Protein to Serum Albumin Ratio; CA-125: Cancer antigen 125; RMI: Risk of Malignancy Index; kg: Kilogram; m: meter; mg: milligram; dL: deciliter; U: unit; ml: milliliter

Characteristics (n=94)	Mean ± SD	Median (IQR)	Range (Min, Max)
Age (Years)	49.29 ± 15.97	51 (42, 59)	73 (12, 85)
BMI (kg/m^2^)	21.79 ± 1.98	22 (20.15, 23)	9.2 (17, 26.2)
C-reactive Protein (mg/dl)	4.50 ± 4.36	3.22 (1.50, 5.85)	21.22 (0.03, 21.25)
Serum Albumin (g/dL)	3.13 ± 0.75	3.2 (2.80, 3.5)	4.68 (0.12, 4.8)
CRP/Alb Ratio	1.60 ± 1.66	0.94 (0.49, 2.19)	8.04 (0.01, 8.05)
CA-125 (U/ml)	1300.01 ± 1314.44	821.50 (429.25, 1678)	5127.8 (32.20, 5160)
RMI	5260.66 ± 7728.67	2397 (551.25, 6073.5)	46425 (15, 46440)

The histopathology of tumors revealed 63.8% (n=60) serous, 19% (n=18) of mucinous, and 17% (n=16) of other types. The surgical staging of the tumors showed 30.9%(n=29) in stage I, 5.3% (n=5) in stage II, 42.6% (n=40) in stage III, and 21.3% (n=20) in stage IV. Ascites were present in 74.5% (n=70) of patients, and metastasis was detected in 59.6% (n=56) of patients.

The median CRP/Alb ratio was 0.94, with a range of 0.01 to 8.05. The serum CA-125 ranged from 32.2 U/ml to 5160 U/ml with a median value of 821.5 U/ml. Both CRP/Alb ratio and CA-125 showed statistically significant association with the clinicopathological parameters (Tables 2-7).

**Table 2:** Association of CRP/Alb Ratio and CA-125 with Age IQR: Interquartile Range; CRP/Alb Ratio: C-reactive Protein to Serum Albumin Ratio; CA-125: Cancer antigen 125; U: unit; ml: milliliter

Variables (n=94)	Age ≤50 Years (n=46) Median (IQR)	Age >50 Years (n=48) Median (IQR)	P value
CRP/Alb Ratio	0.89 (0.44, 1.93)	0.94 (0.49, 2.61)	0.571
CA-125 (U/ml)	839.50 (447.50, 1582.65)	779.50 (372.25, 1719.17)	0.973

**Table 3:** Association of CRP/Alb Ratio and CA-125 with Metastasis IQR: Interquartile Range; CRP/Alb Ratio: C-reactive Protein to Serum Albumin Ratio; CA-125: Cancer antigen 125; U: unit; ml: milliliter

Variables (n=94)	Metastasis Present (n=56) Median (IQR)	Metastasis Absent (n=38) Median (IQR)	P value
CRP/Alb Ratio	1.43 (0.56, 2.83)	0.78 (0.30, 1.07)	<0.001
CA-125 (U/ml)	1520.00 (701.00, 2549.20)	470.50 (121.75, 654.00)	<0.001

**Table 4:** Association of CRP/Alb Ratio and CA-125 with Lymph Node Involvement IQR: Interquartile Range; CRP/Alb Ratio: C-reactive Protein to Serum Albumin Ratio; CA-125: Cancer antigen 125: U: unit; ml: milliliter

Variables (n=94)	Lymph Node Involvement Present (n=61) Median (IQR)	Lymph Node Involvement Absent (n=33) Median (IQR)	P value
CRP/Alb Ratio	1.47 (0.68, 2.82)	0.51 (0.26, 0.93)	<0.001
CA-125 (U/ml)	1500.00 (722.00, 2190.00)	431.00 (109.00, 573.50)	<0.001

**Table 5:** Association of CRP/Alb Ratio and CA-125 with Tumor Grade IQR: Interquartile Range; CRP/Alb Ratio: C-reactive Protein to Serum Albumin Ratio; CA-125: Cancer antigen 125: U: unit; ml: milliliter

Variables (n=94)	High Tumor Grade (n=62) Median (IQR)	Low Tumor Grade (n=32) Median (IQR)	P value
CRP/Alb Ratio	1.46 (0.63, 2.82)	0.52 (0.25, 0.94)	<0.001
CA-125 (U/ml)	1478.30 (679.00, 2161.45)	427.50 (104.50, 594.25)	<0.001

**Table 6:** Association of CRP/Alb Ratio and CA-125 with Tumor Stage (FIGO Staging System) IQR: Interquartile Range; CRP/Alb Ratio: C-reactive Protein to Serum Albumin Ratio; CA-125: Cancer antigen 125: FIGO: International Federation of Gynecology and Obstetrics; U: unit; ml: milliliter

Variables (n=94)	Stage-I (n=29) Median (IQR)	Stage-II (n=05) Median (IQR)	Stage-III (n=40) Median (IQR)	Stage-IV (n=20) Median (IQR)	P value
S CRP Albumin Ratio	0.49 (0.25, 0.90)	0.95 (0.90, 2.30)	1.47 (0.56, 2.83)	1.58 (0.88, 3.06)	<0.001
CA-125 (U/ml)	431.00 (120.50, 573.50)	473.00 (80.70, 665.50)	1321.25 (677.00, 1926.75)	1715.45 (1010.25, 3050.75)	<0.001

**Table 7:** Association of CRP/Alb Ratio and CA-125 with Ascites IQR: Interquartile Range; CRP/Alb Ratio: C-reactive Protein to Serum Albumin Ratio; CA-125: Cancer antigen 125; U: unit; ml: milliliter

Variables (n=94)	Ascites Present (n=70) Median (IQR)	Ascites Absent (n=24) Median (IQR)	P value
CRP/Alb Ratio	1.16 (0.63, 2.76)	0.46 (0.25, 0.88)	<0.001
CA-125 (U/ml)	1321.25 (518.82, 1984.97)	460.50 (119.25, 594.25)	<0.001

**Figure 1:**
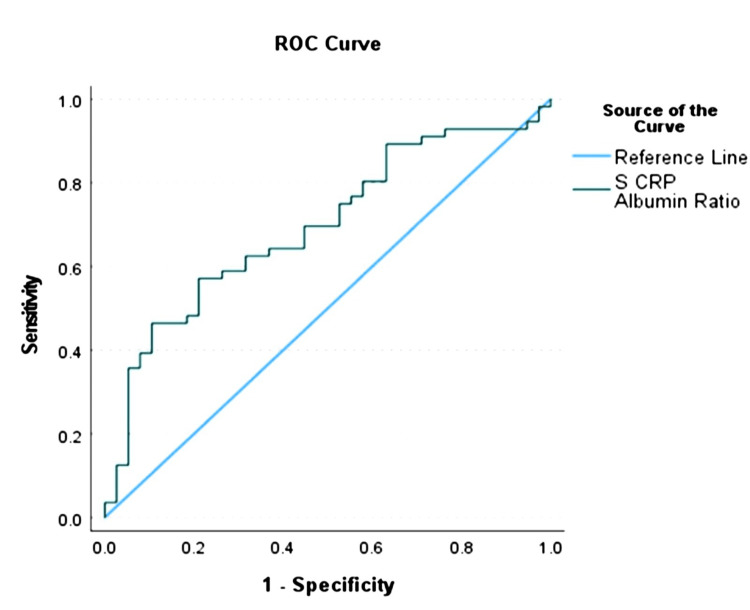
Receiver operating characteristic (ROC) graph (CRP/Alb Ratio) S CRP: Serum C-Reactive Protein; Alb: Serum Albumin

For the CRP/Alb ratio, an optimal cut-off value of 1.08 with a 95% confidence interval (CI) of 0.59 to 0.8 was calculated using the presence of metastasis as the endpoint (Table 8, Figure 1).

**Table 8:** ROC Analysis to Determine the Optimal Cut-off Points for CRP/Alb Ratio in Predicting Metastasis AUC: Area Under Curve, CI: Confidence Interval, %: Percentage, P<0.05: Statistical Significance

AUC	95% CI	Cut-off Value	Sensitivity	Specificity	P value
0.70	(0.59, 0.80)	1.08	57.1%	78.9%	<0.001

**Figure 2:**
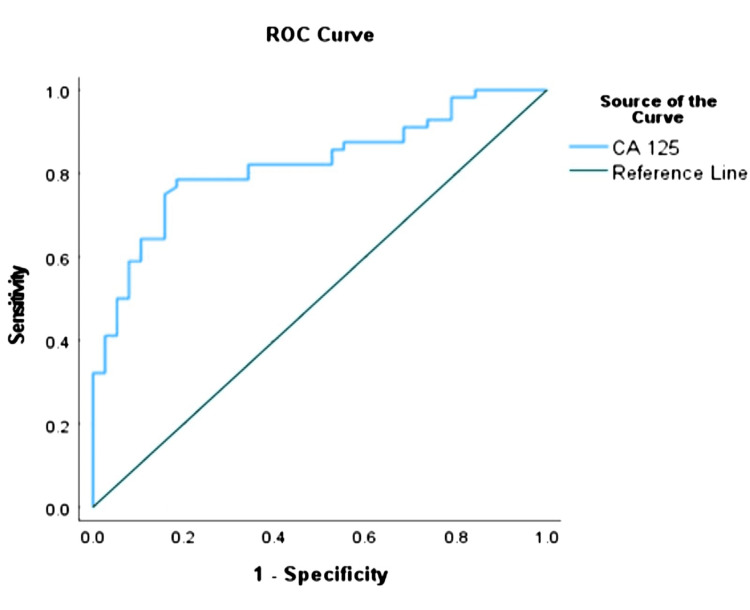
Receiver operating characteristic (ROC) graph (CA 125) CA 125: Cancer Antigen 125

Similarly, the optimal cut-off value for CA-125 was 671.5 (95% CI of 0.74-0.91) using the same endpoint (Table 9, Figure 2).

**Table 9:** ROC Analysis to Determine the Optimal Cut-off Points for CA-125 Ratio AUC: Area Under Curve, CI: Confidence Interval, %: Percentage, P<0.05: Statistical Significance

AUC	95% CI	Cut-off Value	Sensitivity	Specificity	P value
0.82	(0.74, 0.91)	671.5	78.6%	81.6%	<0.001

Based on the cut-off point, 40 patients had a high (≥1.08), and 54 patients had a low CRP/Alb ratio (<1.08). The patients with elevated CRP/Alb ratio (≥1.08) had significantly increased chances of higher tumor stage (p<0.001), lymph node involvement (p<0.001), presence of ascites (p=0.004), higher serum CA-125 (p=0.002), and higher tumor grade (p<0.001) (Table 10).

**Table 10:** Categorization of Patients Based on Cutoff Value of CRP/Alb Ratio (1.08) and Their Association with Clinicopathological Variables CRP/Alb Ratio: C-reactive Protein to Serum Albumin Ratio; CA-125: Cancer antigen 125; FIGO: International Federation of Gynecology and Obstetrics; n: Numbers, %: Percentage; U: unit; ml: milliliter

Variables (n=94)	CRP/Alb Ratio (<1.08) (n=54)	CRP/Alb Ratio (≥1.08) (n=40)	P value
CA-125 (U/ml)			
CA-125 (<671.5)	32 (59.3%)	11 (27.5%)	0.002
CA-125 (>=671.5)	22 (40.7%)	29 (72.5%)	
Metastasis			
Present	24 (44.4%)	32 (80.0%)	<0.001
Absent	30 (55.6%)	8 (20.0%)	
Ascites			
Yes	34 (63.0%)	36 (90.0%)	0.004
No	20 (37.0%)	4 (10.0%)	
Tumor Stage (FIGO)			
Stage-I	25 (46.3%)	4 (10.0%)	<0.001
Stage-II	4 (7.4%)	1 (2.5%)	
Stage-III	18 (33.3%)	22 (55.0%)	
Stage-IV	7 (13.0%)	13 (32.5%)	
Histological Type			
Serous	30 (55.6%)	30 (75.0%)	0.102
Mucinous	14 (25.9%)	4 (10.0%)	
Others	10 (18.5%)	6 (15.0%)	
Lymph Node Involvement			
Yes	25 (46.3%)	36 (90.0%)	<0.001
No	29 (53.7%)	4 (12.1%)	
Tumor Grade			
Low	28 (51.9%)	4 (10.0%)	<0.001
High	26 (48.1%)	36 (90.0%)	

## Discussion

This single-center study investigated the association between the preoperative CRP/Alb ratio and clinicopathological characteristics in 94 histopathologically confirmed ovarian cancer patients (aged 12-85). Patients with distant metastasis, lymph node involvement, ascites, higher tumor grade, and advanced stages (III and IV) exhibited significantly higher median CRP/Alb ratios. These same parameters correlated with elevated CA-125 levels. Receiver operating characteristic (ROC) analysis determined optimal cutoff values of 1.08 for the CRP/Alb ratio and 671.5 Units/ml for CA-125, using metastasis as the endpoint. A preoperative CRP/Alb ratio above 1.08 strongly indicated a significantly higher likelihood of severe disease and poor clinicopathological features.

This study builds upon previous research. Liu et al., in a retrospective study (n=200), investigated the association of various inflammatory markers, including CRP/Alb ratio, with clinicopathological parameters [17]. This study identified a CRP/Alb ratio cutoff of 0.68, predictive of overall survival. Ratios above this threshold were significantly associated with poorer survival outcomes and also correlated with advanced cancer stage, lower rates of successful cytoreductive surgery, the presence of ascites, and elevated CA-125 levels. Komura et al., in another retrospective study (n=307), also showed elevated CRP/Alb ratios in patients with more advanced clinical stages, ascites, and higher CA-125 levels, identifying cutoff values of 0.7793 (disease-specific death) and 0.048 (survival) [18]. Univariate and multivariate analyses confirmed the CRP/Alb ratio as an independent prognostic factor for shorter disease-specific survival. A meta-analysis to investigate the role of CRP/Alb ratio in gynecological cancers by Fang et al. confirmed that a high pretreatment CRP/Alb ratio correlated with poor overall and progression-free survival and stage III-IV disease [19]. However, the associations with lymph node metastasis, tumor size, and histopathological grade were not significant in their analysis. Recently, a prospective study (n=69) by Budiana et al. also found higher median CRP/Alb levels in advanced-stage ovarian cancer patients (11.0 vs 1.92; p=0.000), calculating a cutoff of 1.34 for diagnosing advanced-stage cancer (sensitivity 86.9%, specificity 68%) [20].

Unlike these previous studies, the current study, due to its shorter duration, did not assess patient survival, focusing instead on the diagnostic utility of the preoperative CRP/Alb ratio in predicting disease severity. The current study's significant association of high CRP/Alb ratio with higher stage, metastasis, ascites, tumor grade, and lymph node involvement contrasts with the Fang et al. meta-analysis. Discrepancies in CRP/Alb cutoff values across studies likely stem from variations in endpoints and patient populations across different geographical locations. This study’s prospective design offers a unique contribution to the field.

The CRP/Alb ratio's advantages include ease of measurement, ready availability, and standardization. Its correlation with aggressive disease phenotypes, as evidenced here and in previous studies, suggests its potential use in early screening, enabling pre-operative treatment modification. Liu et al.'s work further suggests its superior prognostic ability compared to other inflammation-based indices, potentially guiding post-treatment follow-up strategies for high-risk patients [17].

This study investigated CA-125 as a routine tumor marker, revealing a strong correlation with disease severity. Significantly, elevated CA-125 levels were also associated with increased CRP/albumin ratios.

Limitations of this study include its single-center design, small sample size, lack of long-term follow-up (precluding survival analysis), and the potential for limited generalizability of the cutoff values. In the future, larger, multicenter studies incorporating diverse patient populations are warranted to validate the findings of the current study.

## Conclusions

This study explored the relationship between preoperative C-reactive protein (CRP) to albumin (Alb) ratio, a readily available inflammatory marker, and clinicopathological characteristics in ovarian cancer patients. The research found a significantly elevated CRP/Alb ratio in patients with advanced disease, defined by the presence of ascites, lymph node involvement, distant metastasis, high tumor grade, and advanced surgical stage. The study determined an optimal cutoff value of 1.08 for the CRP/Alb ratio. Patients with a preoperative CRP/Alb ratio exceeding this threshold demonstrated a substantially increased likelihood of exhibiting these unfavourable clinicopathological features. Furthermore, elevated CA-125 levels were also observed in patients with advanced disease and significantly associated with increased CRP/Alb ratio. In essence, a higher CRP/Alb ratio before surgery was associated with a more severe and advanced stage of ovarian cancer. Therefore, the preoperative CRP/Alb ratio is proposed as a valuable addition to routine tumor marker assessment in ovarian cancer.

## Appendices

**Table 11:** Patient Recruitment Questionnaire/Proforma CA 125: Cancer antigen 125; FIGO: International Federation of Gynecology and Obstetrics; CT Scan: Computed Tomography Scan

Serial No.	
Name	
Patient Identity No.	
Address with contact details	
Age (Years)	
Parity	
Height (cm)	
Weight (Kg)	
Presenting complaints at the time of diagnosis	
Menstrual status: Premenopausal/Postmenopausal	
Comorbidities if Any:	
History of polycystic ovarian syndrome (PCOS): Yes/ No	
History of Endometriosis	
History of infertility: Yes/ No	
History of Breast feeding: Yes/ No	
History of other malignancy: Yes/ No	
Family history of Malignancy: Yes/ No; If Yes Type	
History of Tubal Ligation: Yes/No	
History of use Oral Contraceptive Pills: Yes/No	
History of Hormone Replacement Therapy: Yes/No	
Addiction: Yes/No; If Yes Type	
Pre-operative serum C-Reactive Protein level	
Pre-operative serum Albumin level	
Pre-operative serum CA125 level	
Ascites: Present/Absent	
Tumor Stage (FIGO staging system): I/II/III/IV	
Histological type: Serous/Mucinous/Others	
Tumor Grade: Low/High	
Lymph Node Metastasis: Yes/No	
Distant Metastasis: Yes/No	
Ultrasound Findings	
CT Scan Abdomen, Pelvis, & Thorax Findings	

## References

[1] Sung H, Ferlay J, Siegel RL, Laversanne M, Soerjomataram I, Jemal A, Bray F (2021). Global Cancer Statistics 2020: GLOBOCAN estimates of incidence and mortality worldwide for 36 cancers in 185 countries. CA Cancer J Clin.

[2] Gangane NM, Patil BU, Ghongade PV (2023). Ovarian cancer: a report from population-based cancer registry at central rural India. J Cancer Res Ther.

[3] Savant SS, Sriramkumar S, O'Hagan HM (2018). The role of inflammation and inflammatory mediators in the development, progression, metastasis, and chemoresistance of epithelial ovarian cancer. Cancers (Basel).

[4] Modugno F, Ness RB, Allen GO, Schildkraut JM, Davis FG, Goodman MT (2004). Oral contraceptive use, reproductive history, and risk of epithelial ovarian cancer in women with and without endometriosis. Am J Obstet Gynecol.

[5] Prizment AE, Folsom AR, Anderson KE (2010). Nonsteroidal anti-inflammatory drugs and risk for ovarian and endometrial cancers in the Iowa Women's Health Study. Cancer Epidemiol Biomarkers Prev.

[6] Clarke SJ, Chua W, Moore M (2011). Use of inflammatory markers to guide cancer treatment. Clin Pharmacol Ther.

[7] Hefler LA, Concin N, Hofstetter G (2008). Serum C-reactive protein as independent prognostic variable in patients with ovarian cancer. Clin Cancer Res.

[8] Allin KH, Nordestgaard BG (2011). Elevated C-reactive protein in the diagnosis, prognosis, and cause of cancer. Crit Rev Clin Lab Sci.

[9] Zhang W, Zhang Z, Qian L (2024). Prognostic and clinicopathological significance of C-reactive protein in patients with ovarian cancer: a meta-analysis. World J Surg Oncol.

[10] Gupta D, Lis CG (2010). Pretreatment serum albumin as a predictor of cancer survival: a systematic review of the epidemiological literature. Nutr J.

[11] Asher V, Lee J, Bali A (2012). Preoperative serum albumin is an independent prognostic predictor of survival in ovarian cancer. Med Oncol.

[12] Ataseven B, du Bois A, Reinthaller A (2015). Pre-operative serum albumin is associated with post-operative complication rate and overall survival in patients with epithelial ovarian cancer undergoing cytoreductive surgery. Gynecol Oncol.

[13] Kinoshita A, Onoda H, Imai N (2015). The C-reactive protein/albumin ratio, a novel inflammation-based prognostic score, predicts outcomes in patients with hepatocellular carcinoma. Ann Surg Oncol.

[14] Liu X, Sun X, Liu J, Kong P, Chen S, Zhan Y, Xu D (2015). Preoperative C-reactive protein/albumin ratio predicts prognosis of patients after curative resection for gastric cancer. Transl Oncol.

[15] Xu XL, Yu HQ, Hu W, Song Q, Mao WM (2015). A novel inflammation-based prognostic score, the C-reactive protein/albumin ratio predicts the prognosis of patients with operable esophageal squamous cell carcinoma. PLoS One.

[16] Wei XL, Wang FH, Zhang DS (2015). A novel inflammation-based prognostic score in esophageal squamous cell carcinoma: the C-reactive protein/albumin ratio. BMC Cancer.

[17] Liu Y, Chen S, Zheng C (2017). The prognostic value of the preoperative c-reactive protein/albumin ratio in ovarian cancer. BMC Cancer.

[18] Komura N, Mabuchi S, Shimura K, Kawano M, Matsumoto Y, Kimura T (2021). Significance of pretreatment C-reactive protein, albumin, and C-reactive protein to albumin ratio in predicting poor prognosis in epithelial ovarian cancer patients. Nutr Cancer.

[19] Fang Y, Zheng T, Zhang C (2021). Prognostic role of the C-reactive protein/albumin ratio in patients with gynecological cancers: a meta-analysis. Front Oncol.

[20] Budiana ING, Mahendra INB, Wiradnyana AAGP, Darmayasa IM, Marta KF, Suwanpramana PM (2023). C-reactive protein/albumin (Crp/Alb) ratio as a predictor of ovarian cancer. Jurnal Health Sains.

